# The impact of COVID-19 stress on nurses’ organizational deviance: A moderated mediation model

**DOI:** 10.1371/journal.pone.0324992

**Published:** 2025-05-28

**Authors:** Zhiyong Han, Mingxia Chen, Yanbo Wang

**Affiliations:** School of Business Administration, Anhui University of Finance & Economics 962, Bengbu, China; The Chinese University of Hong Kong, HONG KONG

## Abstract

The outbreak and rapid spread of the COVID-19 in December 2019 (Iqbal Z, Aslam MZ, Aslam T, Ashraf R, Kashif M, Nasir H, Register J, 2020, 13, 208–30) has brought great work pressure to nurses on the frontline of the fight against the virus, which is very likely to lead to work deviant behaviors, therefore, how to effectively manage nurses to inhibit their organizational deviance in the context of an emergency public health crisis has a high research value. A questionnaire was administered to 319 Chinese in-service nurses, and SPSS and AMOS software were used to conduct correlation analysis, confirmatory factor analysis, and hierarchical regression analysis to statistically test the hypotheses of the developed model. COVID-19 stress can significantly positively predict nurses’ organizational deviance. The relationship between the two variables is mediated by job satisfaction. Furthermore, perceived organizational support(POS) demonstrates a dual moderating function in our framework: it not only influences the relationship between CST and employee job satisfaction, but also affects the extent to which satisfaction mediates subsequent organizational outcomes. COVID-19 stress is an important psychological factor influencing nurses’ organizational deviance. The government and relevant organizations are supposed to take the psychological stress of such primary medical staff seriously, provide more supportive resources and take various measures to reduce COVID-19 stress to help individuals cope with the COVID-19 crisis.

## Introduction

COVID-19 broke out and spread rapidly in December 2019, which not only seriously endangered the safety of public life and property but also had a severe impact on human psychology and caused enormous difficulties in centralized treatment [1,2]. Nurses, who serve on the frontlines of the fight against the COVID-19 epidemic, are a crucial force in the rescue team, and they not only are subjected to high-intensity, high-risk work but also face tremendous physical and psychological stress [3]. Recent studies have highlighted that healthcare workers, particularly nurses, continue to experience substantial psychological consequences even after the acute phases of the pandemic [4,5].Under such circumstances, nurses who have been under stress for a long period of time may experience adverse emotional responses such as anxiety, depression, job burnout, and resignation intention [6–8]. Long-term stress accumulation leaves nurses highly prone to deviant behavior [9,10], creating new challenges for global public health as well as research and medical communities. Therefore, it is vital to actively explore the mechanism underlying nurses’ deviant organizational behavior to regulate their behaviors, reduce disputes and violence, and provide high-quality medical care services [11]. This is an important research direction under the current context.

Organizational deviance (OD) is defined as employees’ spontaneous violation of important organizational norms, which undermines the interests of the entire organization [12]. It is an important dimension of workplace deviance, with common examples including late arrival and early departure. OD can pose many potential threats, resulting in low individual productivity and, in severe cases, nursing errors and potential safety hazards. To date, few studies have been conducted on nurses’ OD and have mainly focused on job demands, perceived organizational fairness, demographic characteristics, and psychological status [13–15].Although some scholars have confirmed that job stress among nurses can positively predict deviant work behavior [15], few researchers have explored the intrinsic mechanism of this kind of stress on nurses’ deviant organizational behavior under the COVID-19 pandemic. It has been proposed that the job stress associated with major public health emergencies tends to cause individuals to consume more emotional resources, experience reduced job satisfaction, and engage in detrimental organizational behavior [16]. In addition, there are individual differences in the destructive power of pandemic-related stress, where perceptions of organizational support are a key factor explaining such differences and a high degree of POS can effectively alleviate the adverse effects of stress [17–19].

Existing literature has several limitations: First, most studies on nurses’ organizational deviance behavior primarily focus on organizational fairness and job demands [13,20], with few studies exploring the impact of stress on nurses’ organizational deviance behavior in the context of public health crises. Second, although research has confirmed that nurses’ work stress can positively predict their work deviation behavior [9], The underlying psychological mechanisms through which COVID-19 stress contributes to organizational deviance among nursing professionals have received limited scholarly attention in the extant literature. Third, individuals differ in their stress responses to major public health crises, but research on the boundary conditions of these differences is insufficient. Finally, Despite its potential utility, the application of Conservation of Resources (COR) theory to examine stress among healthcare professionals remains understudied in the scholarly literature, with a particularly significant gap evident in crisis-related contexts.

This investigation seeks to develop and validate a moderated mediation model to examine the relationship mechanism between COVID-19 stress and nurses’ organizational deviance behavior. Specifically, this study aims to: (1) explore how COVID-19 stress directly affects nurses’ organizational deviance behavior; (2) test the mediating role of job satisfaction in the relationship between COVID-19 stress and organizational deviance behavior; and (3) analyze the moderating role of POS in the relationship between COVID-19 stress and job satisfaction.

To this end, we develop a moderated mediation model by collecting data from Chinese in-service nurses to examine the impact and mechanism of perceived COVID-19 stress (CST) on Chinese nurses’ OD. There are the following contributions to this present study: First, Our study extends the literature the antecedents of nurses’ OD in crisis situations by identifying COVID-19 stress as a critical factor influencing deviant behaviors among nursing staff, thereby extending previous organizational deviance literature that primarily focused on organizational fairness and job demands. Second, we deepen the study on the mechanism underlying the impact of perceived stress on individual behavior in crisis contexts by introducing job satisfaction as a crucial mediating variable, which helps explain how and why COVID-19 stress translates into organizational deviance. Third, we examine the moderating role of POS in the model and explore the boundaries of the impact of CST on nurses’ OD through perceived satisfaction, providing empirical evidence for the importance of organizational support in mitigating the negative effects of COVID-19 stress. This study provides a theoretical basis for the development of intervention measures for psychological problems experienced by Chinese nurses within the COVID-19 period as well as practical guidance for reducing nurses’ deviant organizational behavior and improving the quality of medical services.

## Theoretical background and hypotheses development

### COVID-19 stress and nurses’ organizational deviance

The prevalence of COVID-19 has brought great challenges to medical staff, who are more likely to be infected and bear more continuous job stress [21,22]. Recent studies indicate that these challenges persist well beyond the initial outbreak phase, with healthcare workers continuing to experience elevated stress levels even as the pandemic evolves [23–25]. Stress can be regarded as an emotional response that occurs when an individual’s needs remain unsatisfied [26,27].Typically, an increase in job stress stimulates organizational members to exhibit negative behaviors [26].The relevant literature suggests that job stress throughout the COVID-19 pandemic has been an important factor influencing nurses’ OD [15,26]. Alagarsamy et al. (2024) found that pandemic-related stressors significantly increase the likelihood of organizational deviance among healthcare personnel, with nurses being particularly vulnerable due to their prolonged patient contact and heightened infection risk [28].

On the one hand, stress itself can lead to deviant behavior. It has been suggested that when employees’ diverse and higher-level needs are not met, deviant organizational behavior such as demotivation, destruction of corporate property, absenteeism, and theft can increase [29]; on the other hand, stress can deplete psychological resources and thus increase the risk of deviant behavior [30]. Existing studies have confirmed that medical workers are more prone to negative psychological problems including anxiety, depression, and job burnout under conditions of sudden stress, which bring distress to individuals’ life and work [31], thereby increasing their detrimental organizational behaviors such as turnover intention and OD [27,32]. Drawing from this analysis, this research posits the following hypothesis

**Hypothesis 1:** CST has a significant positive impact on nurses’ OD.

### The mediating role of job satisfaction

Hoppock et al. (1938) argues that job satisfaction refers to employees’ feeling of satisfaction with their work situation in both psychological and physical respects [33].Among various influencing factors, job stress has the most direct and immediate impact on job satisfaction [34]. Scholars have established that excessive job stress can reduce nurses’ job satisfaction, which in turn negatively affects individual job performance [35,36]. Nurses have constantly been at risk throughout the COVID-19 pandemic due to heavy workloads, understaffing, chronic fatigue, the threat of infection, and frustration with patient deaths, causing greater psychological stress [37]. When subjectively perceived stress increases, nurses’ negative emotions become more pronounced. If nurses cannot actively regulate and transform these negative emotions, they can further exacerbate individuals’ negative perceptions and evaluations of their jobs and lead to a decrease in job satisfaction [38].

Among the key attitudinal constructs examined in organizational research, job satisfaction emerges as a particularly robust predictor of employees’OD, demonstrating significant explanatory power in workplace behavior models [39]. When individuals become dissatisfied with their jobs, they will develop a strong willingness to act in deviant ways of intentionally being late or taking long breaks to hinder organizational performance and offset unsatisfactory conditions encountered at work (e.g., low pay or a lack of promotion opportunities).

Conservation of resources (COR) theory provides a robust theoretical framework for understanding the relationship between COVID-19 stress and organizational deviance [40]. COR theory holds that the potential depletion or actual loss of any resource poses a threat to individuals and impedes the occurrence of positive behaviors [41]. According to this theory, individuals strive to obtain, retain, and protect resources, and they experience stress when resources are threatened, lost, or when investment of resources does not result in adequate return [42]. In the context of the COVID-19 pandemic, nurses experience significant resource depletion due to increased workload, infection risk, and emotional strain [43,44]. Nurses’ coping with CST is premised on consuming resources. When individuals cannot obtain sufficient resources to relieve stress in a timely manner, they easily form negative cognitive evaluations and emotional responses to their work, experience reduced job satisfaction, and consequently engage in more negative behavior.

**Hypothesis 2:** Job satisfaction mediates the relationship between CST and nurses’ OD.

### The moderating role of perceived organizational support

Organizational support is a concept proposed by Eisenberger et al. (1986) based on reciprocity norms and social exchange theory [45]. It is a form of social support and refers to employees’ feeling care, support, and recognition from their organizations. Social support, as an important psychological resource, has been sought by individuals when perceiving stress from the COVID-19 pandemic, thereby helping individuals reduce experiences of cognitive and emotional depletion [46]. Previous research findings indicate that organizational social support effectively attenuates the relationship between stressors and their adverse consequences within the context of the COVID-19 pandemic [19,47]. Hebles et al. (2022) reveal that in the context of the COVID-19 pandemic, the detrimental impact of perceived stress on psychological safety is weakened when employees of healthcare organizations feel supported by their senior supervisors [48]. Zhou et al. (2022) also confirm that during the prevention and control of major infectious diseases [49], POS significantly negatively moderates the direct relationship of job stress with depression and anxiety as well as the relationship between work stress and burnout among medical workers. Based on this evidence, we contend that POS functions as a significant contextual variable that shapes the effectiveness of CST interventions for nursing personnel. Employees who report high levels of POS tend to exhibit enhanced positive emotional states and favorable workplace experiences, thus helping replenish resources depleted by stress and buffering against the effects of negative emotions or stress caused by the depletion of their own resources [50,51]. In contrast, the perceived job stress of primary medical staff with low levels of POS is more likely to exacerbate a reduction in job satisfaction [52,53].Accordingly, we propose that:

**Hypothesis 3:** POS moderates the relationship between CST and nurses’ job satisfaction. Specifically, the lower the level of POS is, the stronger the negative impact of CST on nurses’ job satisfaction is and vice versa.

Drawing upon Hypotheses 2 and 3, our analysis demonstrates that job satisfaction functions as a mediating variable, while POS exerts a moderating influence on the relationship between CST and nurses’ job satisfaction. These two hypotheses provide a theoretical basis for a mediated mediation model, with POS acting as a boundary condition for the indirect mechanism underlying the impact of CST on deviant organizational behavior. We suggest that when low levels of POS are, the stronger the impact of CST on nurses’ OD through job satisfaction is, and vice versa. Accordingly, we propose the following hypothesis:

**Hypothesis 4:** The mediating function of job satisfaction in the relationship between CST and nurses’ OD is expected to be negatively moderated by POS, whereby the indirect effect strengthens as nurses’ POS diminish.

**Fig 1:**
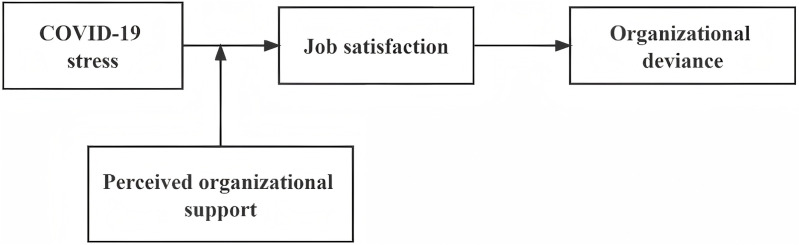
The conceptual research model.

This study attempt to examine on the mediating effect of job satisfaction and the moderating effect of POS in the relationship between nurses’ CST and OD. The research results are conducive to providing a theoretical basis and practical guidance for improving the service quality of nurses in public health emergencies. In order to understand the research content more intuitively, the following conceptual model is constructed as shown in Fig 1.

## Method

### Design and sample

We conducted a cross-sectional, self-reported study of Chinese nurses’ organizational deviance and strictly followed the guidelines for reporting observational studies(STROBE-Strengthening the Reporting of Observational Studies in Epidemiology [54]). This methodological approach was selected based on its appropriateness for examining relationships between psychosocial variables in workplace settings [55,56].

We first designed the questionnaire using WJX, a popular online questionnaire survey platform in China, and then distributed it through numerous chat applications such as WeChat. Due to the necessity of adhering to public health guidelines during the COVID-19 outbreak, a web-based questionnaire methodology was implemented to facilitate expedient data acquisition while eliminating physical proximity requirements [57,58]. The survey design followed established guidelines for online health research [59], including clear instructions, appropriate question sequencing, and mobile optimization.

We then selected nursing managers from two large hospitals in eastern and southern China to help us distribute the survey. Data acquisition was conducted between December 2022 and January 2023, coinciding with a period when the epidemic exhibited high prevalence rates. The questionnaire was filled out anonymously and voluntarily during the data collection process, and the confidentiality of responses was guaranteed.

A total of 320 questionnaires were recovered through online data investigation. After excluding 20 questionnaires due to duplication, incompleteness, completion within an extremely short time, or logically inconsistent responses, 300 valid questionnaires were finally obtained, resulting in a validity rate of 93.75%. Among the participants, 6.7% were male and 93.3% were female, the vast majority (72.0%) of nurses in the surveyed sample had a bachelor’s degree, most (46.7%) of the nurses were aged 30–39, and most (33.7%) of them had worked for 11–20 years.

### Validity and reliability

First, we conducted a pilot test with 20 nurses to assess the clarity and relevance of questionnaire items, resulting in minor adjustments to wording and formatting [60]. Second, to verify the internal consistency reliability of our measurement instruments, Cronbach’s alpha coefficients were computed for each scale. Third, Confirmatory factor analysis(CFA) was employed to assess the construct validity and discriminant validity among the study variables. Finally, we employed procedural remedies to minimize common method bias(CMB), including protecting respondent anonymity, varying response formats, and Stressing the absence of correct or incorrect responses [61].

### Measures

To confirm the reliability and validity of the employed scales, we chose scales that are frequently used and have been published in high-quality journals for measuring the main variables included in this study. In addition, to guarantee the relevance of these English measurement instruments within the Chinese cultural context, we implemented a rigorous translation and back-translation procedure for all scales. Each instrument employed a five-point Likert format, ranging from 1 (strongly disagree) to 5 (strongly agree).

*COVID-19 stress.* This scale was adapted from the 14-item stress scale compiled by Cohen et al. (1983) [62], removing the items which do not relevant to the nursing work situation. Finally, the remaining six items were used to measure nurses’ CST with a representative item being “I feel very unsafe because of the COVID-19 pandemic.” The Cronbach’s alpha was 0.913.

*Organizational deviance.* We used a three-item scale developed by Decells et al. (2012) [63], with a representative item being “I have intentionally left work early.” In this study, the Cronbach’s alpha was 0.942.

*Job satisfaction.* We used a three-item scale developed by Liu et al. (2007) [64], with a representative item being “Generally speaking, I do not like my job.” In this study, the Cronbach’s alpha was 0.877.

*Organizational support.* We used a simplified eight-item scale developed by Eisenberger et al. (2002) which is currently the most popular and widely used tool for measuring POS [65]. As suggested by Hair et al. (2010) [66], items with low factor loadings were removed, and seven items were retained, including “My organization cares about my opinion.” the Cronbach’s alpha was 0.895.

*Control variables.* Previous researches have shown that various demographic factors of participants, such as gender, age, and other individual factors, may impact employees’ deviant behavior [67,68]. In the comprehensive model, demographic factors such as gender, age, educational level, and work tenure were controlled for to minimize confounding effects on the primary research variables’ relationships.

### Data analysis

Data analysis proceeded through several systematic steps. First, Using SPSS 26.0, we began with preliminary analyses that encompassed cleaning the dataset, testing for normality, and examining both descriptive statistics and bivariate correlations. Second, To evaluate the measurement model’s fit and establish discriminant validity among the constructs, CFA was conducted using AMOS 24.0. Third, we tested the direct effect of COVID-19 stress on organizational deviance using hierarchical regression analysis. Fourth, we examined the mediating role of job satisfaction using the bootstrapping procedure with 5000 samples [69]. Fifth, Through an analysis of interaction terms, we examined the moderating effect of POS. Finally, we integrated these findings to test the moderated mediation model using PROCESS macro (Model 7) with bootstrapping [69,70].

## Results

### Confirmatory factor analysis

Using AMOS software, we conducted a CFA on our collected data to examine the discriminant validity of four key latent variables (CST, POS, job satisfaction, and OD) by constructing and comparing one-, two-, three-, and four-factor models. The output results are shown in Table 1. A comparison of the following types of models reveals that the fitting effect of the 4-factor model is optimal and that all indicators are within a reasonable range (χ^2^/df = 2.213, RMSEA = 0.064, CFI = 0.959, TLI = 0.951, SRMR = 0.075). Therefore, the discriminant validity of these variables included in the research is good. To assess convergent validity, we examined the standardized factor loading coefficients, calculated the composite reliability (CR), and determined the average variance extracted (AVE). The running results show that the standardized factor loadings of the four-factor measurement items are above the critical value of 0.5. The variables have CRs in the range of 0.885 to 0.986, which are higher than 0.8, and AVEs in the range of 0.543 to 0.848, which are greater than 0.5, indicating the good convergent validity of these variables.

**Table 1:** Confirmatory factor analysis results.

Model	χ²	df	χ²/df	CFI	TLI	RMSEA	SRMR
4-factor model: CST; POS; JS; OD	314.226	142	2.213	0.959	0.951	0.064	0.075
3-factor model: CST; POS + JS; OD	733.024	149	4.920	0.862	0.841	0.114	0.076
2-factor model: CST + POS + JS; OD	1601.097	151	10.603	0.657	0.611	0.179	0.141
1-factor model: CST + POS + JS + OD	2423.056	152	15.941	0.462	0.395	0.224	0.172

*Note:* + : Two factors were combined.

Abbreviations: CST, COVID-19 stress; POS, perceived organizational support; JS, job satisfaction; OD: organizational deviation.

### Common method bias analysis

Since data were collected through employee self-rating, CMB was unavoidable despite the design by specifying the research purpose, emphasizing the privacy of information obtained, and using reverse items for control beforehand. To ensure the rigor of the data, CMB was tested using Harman’s one-factor test. The results show that without data rotation, we finally extract four factors with eigenvalues greater than 1, and the variation explained by the first factor is 39.15% which is less than 40%, indicating that the problem of CMB was not too serious. Following Podsakoff et al. (2003) [71], we reevaluated our measurement model by implementing the unmeasured latent factor approach to assess potential CMB. The differences in the fitting indices between the original model and this model with common factors (△CFI = 0.005, △TLI = 0.001, △SRMR = 0.033, and △RMSEA = 0.001) are within reasonable ranges. The variation of the fit index RMSEA does not exceed 0.05, and the variations of fit indices CFI and TLI are less than the criterion of 0.05 [72], suggesting that the CMB for the collected data is not severe.

### Descriptive statistics and correlation analysis

The means, standard deviations, and correlation coefficients of the important variables of this paper are shown in Table 2. The results indicate a significant negative correlation between CST and employee job satisfaction (r = −0.396, p < 0.01) and significantly positively correlated with OD (r = 0.158, p < 0.01). Furthermore, there was a significant negative relationship between job satisfaction and OD (r = −0.227, p < 0.01). The above results provide preliminary support for the verification of the hypothesis.

**Table 2:** Descriptive statistics and correlations.

Measure	Mean	SD	1	2	3	4	5	6	7
1.Gend	1.933	0.250							
2.Edu	2.740	0.483	0.105						
3.Age	2.910	0.958	0.156**	0.043					
4.Tenur	3.487	1.126	0.235**	0.061	0.865**				
5.CST	2.811	0.819	-0.130*	-0.015	0.012	0.035			
6.POS	3.448	0.711	0.158**	-0.009	0.122*	0.095	-0.387**		
7.JS	3.702	0.795	0.062	0.036	0.143*	0.140*	-0.396**	0.705**	
8.OD	1.456	0.675	-0.070	-0.018	-0.069	-0.098	0.158**	-0.170**	-0.227**

*Note: N* = 300. * *p* < 0.05; ** *p* < 0.01.

Abbreviations: CST, COVID-19 stress; POS, perceived organizational support; JS, job satisfaction; OD, organizational deviation.

### Hypothesis testing

To examine the proposed hypotheses, we conducted hierarchical regression analyses using SPSS 26.0 [73], and sort out data results in Table 3 are as follows. In Model 4, the control and independent variables are successively placed in the regression model, and the results of running the data show that after accounting for the control variables, CST can significantly positively predict nurses’ OD (β = 0.159, p < 0.01), thereby supporting H1. When both CST and job satisfaction variables are incorporated together in our regression analysis, as shown by Model 5, job satisfaction can significantly negatively affect OD (β = −0.184, p < 0.01), while the correlation between CST and OD is no longer significant (the β value decreases to 0.085, and p > 0.05), suggesting that job satisfaction fully mediates the relationship between CST and organizational deviance. Thus, H2 was supported.

**Table 3:** Multiple linear regression analysis results.

Variables	JS			OD	
	M1	M2	M3	M4	M5
1. Gender	-0.027	-0.092*	-0.092*	-0.023	-0.028
2. Education	0.023	0.042	0.045	-0.007	-0.003
3. Age	0.054	-0.044	-0.041	0.068	0.078
4. Tenur	0.112	0.141	0.138	-0.156	-0.136
CST	-0.404***	-0.160***	-0.150**	0.159**	0.085
JS					-0.184**
POS		0.650***	0.659***		
CST × POS			0.087*		
R^2^	0.183	0.531	0.538	0.037	0.065
△R^2^	0.159	0.507	0.515	0.025	0.052
F	13.139***	55.238***	48.614***	2.287*	3.402*

*Note:* *p < 0.05, **p < 0.01.

Abbreviations: CST, COVID-19 stress; POS, perceived organizational support; JS, job satisfaction; OD, organizational deviation.

Following the suggestions of MacKinnon et al. (2004) [74],we further tested the mediating effect of job satisfaction using the PROCESS plug-in in SPSS with 5000 bootstrap repeated samples and a confidence interval (CI) level set to 95%. From Table 4, we can see that the mediating effect of job satisfaction has a value of 0.0613, with 95%CI=[0.018, 0.121], which does not include 0. The direct effect between CST and OD has a value of 0.070, with 95%CI=[−0.031, 0.172], including 0. The above results imply that job satisfaction fully mediated the relationship between CST and OD, further supporting H2.

**Table 4:** Standardized indirect effects.

	Effect	se	t	p	LLCI	ULCI
Total	0.132	0.048	2.752	0.006	0.037	0.226
Direct	0.070	0.052	1.360	1.360	-0.031	0.172
Indirect	0.061	0.027	/	/	0.018	0.121

**Fig 2:**
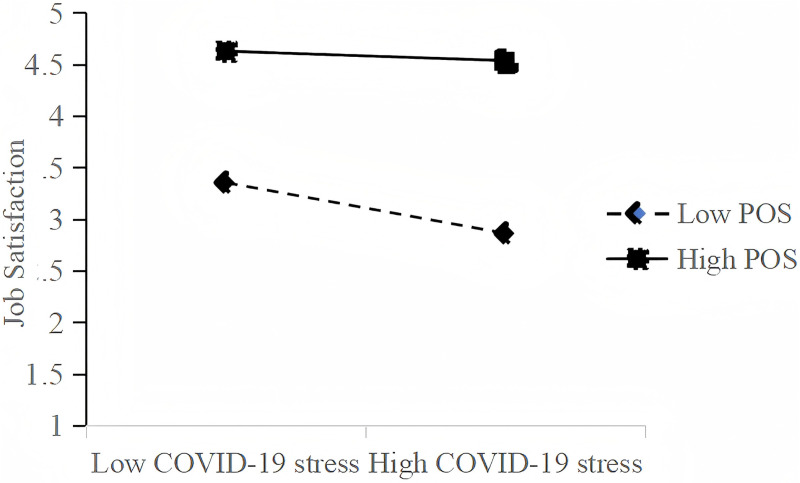
Moderating effect of POS on the relationship between COVID-19 stress and job satisfaction. Abbreviations: POS, perceived organizational support.

For the moderating effect of POS, Model 3 (shown in Table 3) shows that after controlling for the main effect of CST and job satisfaction, the interaction item of CST and POS can significantly positively affect job satisfaction (β = 0.087, p < 0.05), indicating the moderating effect of POS. Furthermore, to better understand the results, we plot the interaction effect of POS using the method developed by Porter and West (1994). The simple slope analysis results show that the negative impact of CST on job satisfaction is exacerbated at low levels of POS (b = −0.217, p < 0.01), and the effect of CST on job satisfaction is not significant at high levels of POS (b = −0.075, p = 0.184). Fig 2 is plotted based on the results and shows that the negative impact of CST on employee job satisfaction is more significant (graphically, the slope is steeper) under low POS than under high POS. Hence, H3 is supported.

For Hypothesis 4, we used the bootstrap method to integrate the mediation and moderation effects into the same analytical framework to verify the moderated mediation model. The analysis results are summarized in Table 5, as shown below. We can see that the indirect effect of CST on nurses’ OD through job satisfaction is significant when employees perceive high levels of organizational deviance (indirect effect = 0.034, 95% CI=[0.008, 0.074]), while the mediating effect of job satisfaction is not significant when nurses perceive low levels of organizational deviance (indirect effect = 0.012, 95% CI=[−0.006, 0.038]). The indirect effects between the cases with high and low levels of POS differ by 0.022, with 95% CI=[0.004, 0.053]). Therefore, H4 is supported.

**Table 5:** Moderated mediation model results.

POS	Effect	Boot SE	Boot LLCI	Boot ULCI
Low POS(-1SD)	0.034	0.017	0.008	0.074
High POS(+1SD)	0.012	0.011	-0.006	0.038
Intergroup differences	0.022	0.013	0.004	0.053

### Findings

Our investigation yielded several significant outcomes, with data analysis confirming that nurses experiencing higher levels of COVID-19-related stress demonstrated increased organizational deviance behaviors (β = 0.32, p < 0.001), thus providing empirical support for our Hypothesis 1. Second, job satisfaction fully mediated the relationship between COVID-19 stress and organizational deviance, with a significant indirect effect (β = 0.15, 95% CI [0.08, 0.23]), supporting Hypothesis 2. Third, In support of Hypothesis 3, our analysis revealed that the relationship between COVID-19 stress and job satisfaction was significantly moderated by POS (β = -0.18, p < 0.01). Finally, Supporting Hypothesis 4, our analysis revealed that POS moderated the relationship between COVID-19 stress and OD as mediated by job satisfaction, Specifically, this conditional indirect effect was more pronounced when employees reported low levels of POS(indirect effect = 0.034, 95% CI [0.008, 0.074]) than when they experienced high levels of POS (indirect effect = 0.012, 95% CI [-0.006, 0.038]).

## Discussion

### Theoretical contributions

There are three aspects of theoretical significance. Firstly, the present work deepens and enriches the study on nurses’ OD. In recent years, the governance of workplace deviant behavior has become increasingly important, and research on this topic in many fields has been of great value [27,75].Previous studies on nurses’ OD have mostly focused on organizational fairness and job demands [13,14]. The present study introduces perceived stress in the crisis of the COVID-19 epidemic to provide a deeper understanding of the factors influencing nurses’ OD.

Secondly, the present work enhances knowledge of the effect and mechanism underlying the impact of CST on nurses under sudden crises such as the epidemic. Amid the COVID-19 epidemic, nurses’ mental health problems have become more pronounced. Previous studies have confirmed relationships between nurses’ perceived stress and anxiety, depression, and turnover intention throughout the COVID-19 epidemic [6,8].However, there has been little research on the mechanisms underlying the psychological impact of OD. For this reason, this paper proposed the mediator of job satisfaction, clarifies the mechanism underlying the role of nurses’ OD. This contribution is particularly significant as it extends the literature on organizational deviance into the context of public health emergencies, which represents a unique and increasingly relevant research setting [76,77].

Finally, our findings contribute to Conservation of Resources theory by demonstrating how resource depletion (through COVID-19 stress) leads to maladaptive behavioral outcomes (organizational deviance), and how resource gain (through organizational support) can buffer this negative process. This extends COR theory applications in healthcare settings and crisis contexts [78,79], providing empirical support for its utility in understanding workplace behaviors during prolonged crises.

### Management implications

First, in view of the negative influence of stress on the working attitudes and behaviors of nurses in the COVID-19 pandemic, hospitals should take the relief and adjustment of job stress among primary nursing staff in sudden major crisis situations seriously to reduce the impact of stress-related factors on employees’ organizational deviance. To this end, nursing managers should formulate appropriate stress management strategies and optimize management systems, such as through regular psychological counseling and lectures, mindfulness, and the popularization and assessment of knowledge about infectious diseases, to help nursing staff alleviate their negative emotions, improve their psychological capital and adaptability, and reduce their job stress.

Second, This findings indicate that job satisfaction serves as a mediating factor through which CST reduces OD among nursing personnel. job satisfaction buffers nurses from CST and thereby affects organizational deviance, and improving job satisfaction helps reduce the negative impact of CST. A positive and pleasant nursing work climate is conducive to improving the job satisfaction of nursing staff and reducing the incidence of nursing errors [80].

Finally, nursing managers should take the inhibiting effect of POS on nurses’ deviant organizational behavior seriously. In strengthening the nurse workforce, hospital managers can make active and effective interventions to improve nurses’ POS, such as by creating a supportive nursing work environment, institutionalizing a reasonable incentive reward and punishment system, and other programs to give nurses more encouragement and support so as to increase their work motivation and autonomy. Providing nurses with more positive psychological resources to protect their mental health improves their job satisfaction, which reduces their OD and allows them to better serve their patients.

## Limitations

Despite the significant theoretical and practical contributions offered by this study, it is necessary to acknowledge several limitations. First, due to the limitations of actual conditions, our selected sample included only in-service nurses of two hospitals in China, which is still not fully representative of all nursing staff, so the generalizability of the results remains to be explored. We, therefore, call for more research in this area, and we may expand the geographic area and scope of our sample to explore in more depth the impact mechanism of nurses’ stress in the context of the COVID-19 pandemic. Second, as our empirical study was only conducted on individuals, our exploration of CST and POS was based on data on nurses’ own perceptions without involving cross-level in-depth research. For this reason, we suggest that multilevel, cross-level research be carried out in the future, as well as hierarchical analysis and research. Finally, as it is impossible to deeply explore causal relationships using a questionnaire, follow-up research can adopt controlled experiments to render our research data more objective and enable more scientific and practical research.

## Conclusion

Within the COVID-19 epidemic, we constructed a mediated moderation model to examine the relationships among CST, job satisfaction, POS, and OD. Taking Chinese in-service nurses as the research object, a model and hypotheses were tested using questionnaire data to empirically analyze the mechanisms underlying the effect of perceived stress on nurses’ psychological states and deviant organizational behavior in the COVID-19 pandemic. Our findings contribute to the literature on organizational behavior and healthcare management by: (1) establishing COVID-19 stress as a significant antecedent of nurses’ organizational deviance; (2) identifying job satisfaction as a key mediating mechanism through which stress influences deviant behavior; (3) identifies POS as a crucial moderating factor that can buffer the adverse impacts of stress stemming from the COVID-19 pandemic; and (4) providing empirical support for the application of Conservation of Resources theory in understanding healthcare workers’ responses to crisis situations.
